# Polymerase theta-helicase promotes end joining by stripping single-stranded DNA-binding proteins and bridging DNA ends

**DOI:** 10.1093/nar/gkac119

**Published:** 2022-03-31

**Authors:** Jeffrey M Schaub, Michael M Soniat, Ilya J Finkelstein

**Affiliations:** Department of Molecular Biosciences and Institute for Cellular and Molecular Biology, The University of Texas at Austin, Austin, TX 78712, USA; Department of Molecular Biosciences and Institute for Cellular and Molecular Biology, The University of Texas at Austin, Austin, TX 78712, USA; Department of Molecular Biosciences and Institute for Cellular and Molecular Biology, The University of Texas at Austin, Austin, TX 78712, USA; Center for Systems and Synthetic Biology, The University of Texas at Austin, Austin, TX 78712, USA

## Abstract

Homologous recombination-deficient cancers rely on DNA polymerase Theta (Polθ)-Mediated End Joining (TMEJ), an alternative double-strand break repair pathway. Polθ is the only vertebrate polymerase that encodes an N-terminal superfamily 2 (SF2) helicase domain, but the role of this helicase domain in TMEJ remains unclear. Using single-molecule imaging, we demonstrate that Polθ-helicase (Polθ-h) is a highly processive single-stranded DNA (ssDNA) motor protein that can efficiently strip Replication Protein A (RPA) from ssDNA. Polθ-h also has a limited capacity for disassembling RAD51 filaments but is not processive on double-stranded DNA. Polθ-h can bridge two non-complementary DNA strands *in trans*. PARylation of Polθ-h by PARP-1 resolves these DNA bridges. We conclude that Polθ-h removes RPA and RAD51 filaments and mediates bridging of DNA overhangs to aid in polymerization by the Polθ polymerase domain.

## INTRODUCTION

DNA double-strand breaks (DSBs) are highly toxic lesions that occur during cellular metabolism and in response to cancer therapies. Non-homologous end-joining (NHEJ)—the predominant DSB repair pathway in human cells—initiates when the ring-like Ku70/80 heterodimer binds the free DNA ends (1,2). Subsequently, Ku recruits additional repair factors to the DSB, including DNA-PKcs to bridge the DNA ends and ligases to seal the break (3,4). Homologous recombination (HR) is an error-free repair pathway that partially processes the free DNA ends to expose 3′-single-stranded DNA (ssDNA) overhangs (5). These overhangs are rapidly bound by replication protein A (RPA). Subsequently, RAD51 replaces RPA on the ssDNA to search for sequence homologies in a sister chromatid (6). RAD51-mediated strand invasion facilitates templated polymerization of a homologous DNA sequence (7). While NHEJ is active throughout the cell cycle, HR is restricted to the S and G2 phases of the cell cycle when a homologous template is available (8,9).

Many cancer types accumulate mutations in NHEJ- or HR-dependent proteins and become reliant on theta-mediated end-joining (TMEJ), an error-prone DSB repair pathway (10). TMEJ is mediated by DNA Polymerase Theta (Polθ), PARP-1 and DNA Ligase III, as well as traditional DNA resection factors (11–13). Unlike HR, TMEJ requires short microhomologies (2–6 bp) and is highly mutagenetic, leading to increased chromosomal rearrangement and short insertions/deletions (14–16). During TMEJ, 3′-ssDNA overhangs are generated by MRE11–RAD50–NBS1 complex (MRN)/CtIP-mediated resection and are annealed at microhomologies (17). The resulting flaps are nucleolytically removed, and Polθ further extends the junctions to stabilize the microhomology (18). However, resected ssDNA is rapidly bound by RPA, which blocks the annealing of microhomologies (19). Furthermore, the recombinase RAD51 displaces RPA with the help of BRCA2 and other recombination mediators (20). Inactivation of TMEJ leads to an increase in HR, suggesting that these two repair pathways are antagonistic (21,22).

**Figure 1.**
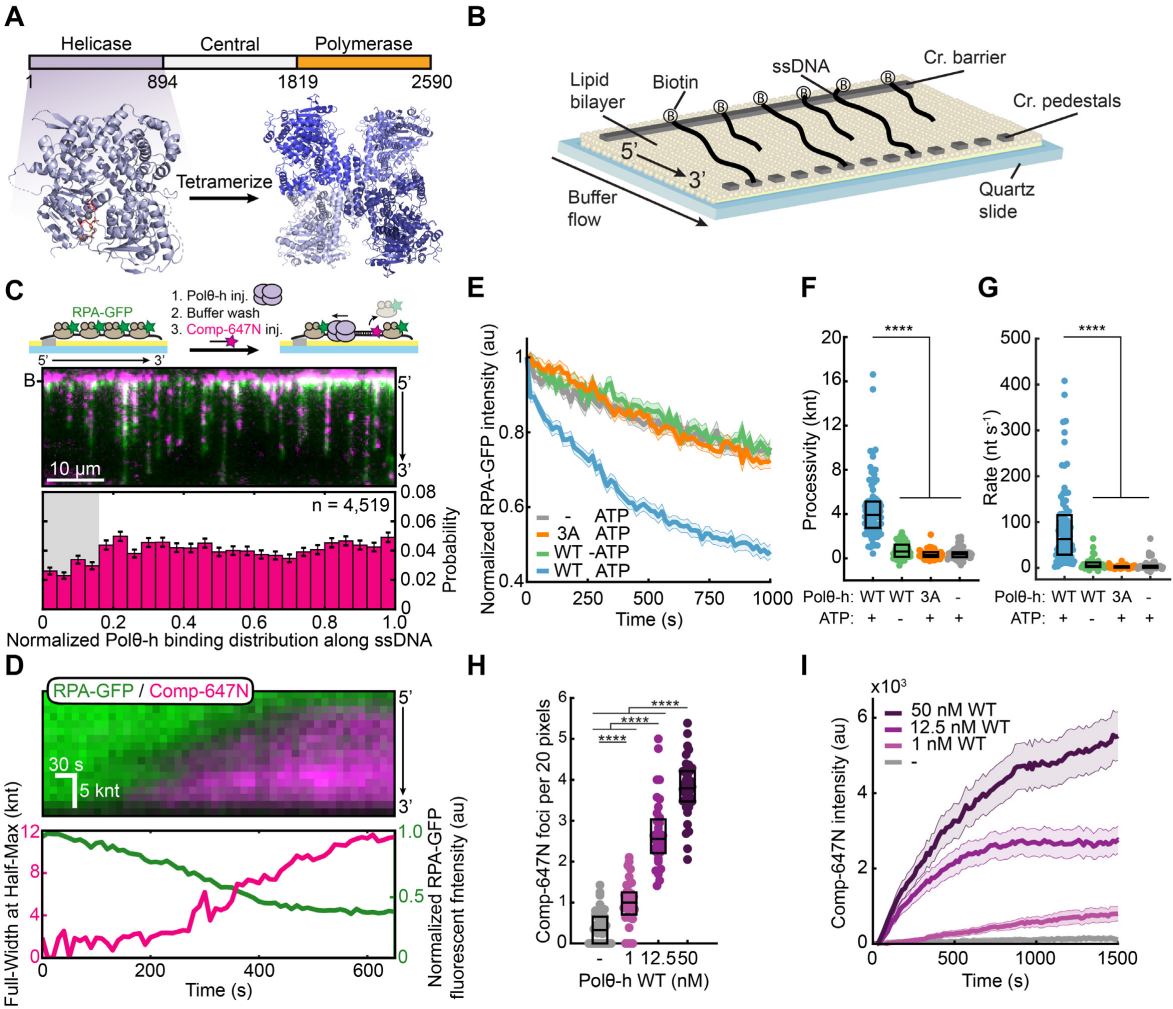
Polθ-h processively removes RPA from single-stranded DNA. (**A**) Polθ domain map (top) and a crystal structure of Polθ-h (PDB: 5A9J). (**B**) Schematic of ssDNA curtains assay. For double-tethered ssDNA curtains, buffer flow is stopped after DNA molecules are immobilized between the chromium barriers and pedestals. (**C**) Schematic (top) and microscope image (middle) of 1 nM Polθ-h stripping RPA-GFP (green) from the ssDNA (top). The cleared regions are marked with a fluorescent complementary oligo (magenta). Histogram of where Polθ-h initiates RPA-GFP removal (bottom). (**D**) (top) Kymograph of processive RPA-GFP (green) removal, as measured by fluorescent complementary oligonucleotide (magenta). Bottom: Analysis of Polθ-h translocation (magenta) and RPA-GFP fluorescence intensity (green). (**E**) Normalized RPA-GFP fluorescent intensity for the indicated experimental conditions. Solid line (average), shading (±SEM). *N* > 46 for all conditions. 1 nM Polθ-h was used, where indicated. (**F**) 1 nM Polθ-h processivity and (**G**) velocity on RPA-coated ssDNA. Box displays median and IQR. *N* > 46 for all conditions. (**H**) Quantification of fluorescent oligonucleotide foci for each Polθ-h concentration on RPA-coated ssDNA. Box displays mean and S.D. (**I**) Fluorescent oligonucleotide intensity across each ssDNA for each Polθ-h concentration. Solid line (average), shading (±SEM).

Polθ is evolutionarily conserved across higher eukaryotes but is missing in fungi (23). Full-length Polθ encodes an N-terminal superfamily 2 (SF2) helicase/ATPase domain, a central disordered domain, and a C-terminal A-family polymerase domain (Figure 1A) (24,25). The isolated Polθ-helicase (Polθ-h) domain is an ssDNA-dependent ATPase that can unwind short DNA duplexes and displace RPA from oligo-length DNA substrates *in vitro* (26,27). In cells, ATPase mutants in the helicase domain increase the prevalence of RAD51 foci after radiation exposure and shift the spectrum of end-joining products with microhomologies near the 3′ ends of DNA substrates (15,21). Polθ is frequently overexpressed in cancers deficient in traditional DSB repair mechanisms, and elevated expression led to poor patient prognosis (28–30). Inhibition of the Polθ-h domain can kill HR-deficient tumor cells, suggesting a therapeutic route for targeting such malignancies (31). Polθ is an especially promising therapeutic target when combined with PARP-1 inhibitors in NHEJ/HR-deficient cancers (21,22,31–33). Together, these studies have established Polθ-h as a critical but enigmatic factor in TMEJ.

Here, we use single-molecule and ensemble biochemical approaches to investigate Polθ-h. Polθ-h is a processive 3′ to 5′ ssDNA-binding motor and can readily displace RPA from ssDNA. Polθ-h can also partially disassemble RAD51 filaments, although this activity is much lower than its ability to remove RPA. Additionally, Polθ-h can bridge two DNA molecules that mimic resection intermediates *in trans* in a reaction that does not require ATP, suggesting that the homotetrameric assembly may tether two arms of a double-strand break during TMEJ. These DNA bridges were resistant to high salt, suggesting additional protein factors may be required for DNA dissociation. Therefore, we investigated the role of PARP-1 regulation of Polθ. PARP-1 rapidly binds to DNA damage sites and initiates the synthesis of poly ADP-ribose (PAR) chains on itself and client proteins that include Polθ. We show that PARP-1 PARylates Polθ-h *in vitro* and reduces the ssDNA binding affinity and promotes dissociation. We conclude that PARP-1 may regulate Polθ-h activity to promote DNA polymerization after the microhomology is established.

## MATERIALS AND METHODS

### Proteins and nucleic acids

Oligonucleotides were purchased from IDT or IBA (for fluorescent oligos) (Supplementary Table S6). Polθ-h (amino acids 1–894) was cloned into a pET19 vector with an N-terminal 6xHis-TwinStrep-SUMO tag to generate pIF378. Polθ-h(3A) mutations E121A, D216A, and E217A were cloned with primers IF733 and IF734 using QuikChange Lightning Multi Site-Directed Mutagenesis Kit to generate plasmid pIF585 (Agilent #210516). Polθ-h(ΔRAD51) replaces residues 861 to 865 with five alanines via inverse PCR with primers IF926 and IF927. RAD51(K133R) was mutagenized using inverse PCR with primers IF724 and IF725. RPA-GFP (plasmid pIF48), RAD51 (plasmid pIF224), and RAD51(K133R) (plasmid pIF582) (34,35).

Polθ-h, Polθ-h(3A), and Polθ-h(ΔRAD51) were purified as described with some modifications (26). Plasmids were transformed into Rosetta(DE3) pLysS (Novagen) *Escherichia coli* cells. Cell pellets were resuspended in Lysis Buffer (25 mM HEPES pH 8.0, 250 mM NaCl, 10 mM imidazole pH 8.0, 5 mM 2-mercaptoethanol, 10% glycerol and supplemented with Roche cOmplete protease inhibitor) and sonicated. The lysed pellet was centrifuged at 40 000 rcf for 45 min. The resulting clarified lysate was placed on a HisTrap column (GE Healthcare) and eluted on a gradient from 10 to 250 mM imidazole. The eluted material was digested with SUMO Protease for 2 h at 4°C and diluted with 25 mM HEPES pH 8.0 to a final NaCl concentration of 100 mM. This was passed through a heparin column (GE Healthcare) and eluted with a gradient from 50 to 1000 mM NaCl. Pure Polθ-h eluted around 600 mM NaCl. Polθ-h-containing fractions were pooled, dialyzed in Dialysis Buffer (25 mM HEPES pH 8.0, 100 mM NaCl, 5 mM DTT and 10% glycerol) for 4 h at 4°C. Polθ-h was spin concentrated and flash-frozen in liquid nitrogen.

PARP-1 was over-expressed from plasmid pIF662 and purified as follows (36). Plasmid pIF662 was transformed into Rosetta(DE3) pLysS (Novagen) *E. coli* cells. Cell pellets were resuspended in Lysis Buffer (25 mM HEPES pH 8.0, 500 mM NaCl, 20 mM imidazole pH 8.0, 0.5 mM 2-TCEP and supplemented with Roche cOmplete protease inhibitor) and sonicated. The lysed pellet was centrifuged at 40 000 rcf for 45 min. The clarified lysate was applied to a HisTrap column (GE Healthcare) and washed with 10 CV of lysis buffer followed by 5 CV of a high salt wash buffer (Lysis Buffer supplemented with 1M NaCl). The column was eluted on a gradient from 20 to 400 mM imidazole. The eluted material was diluted with 25 mM HEPES pH 8.0 to a final NaCl concentration of 100 mM. This was passed through a heparin column (GE Healthcare) and eluted with a gradient from 50 to 1000 mM NaCl. Eluted PARP-1 was concentrated to ∼1 ml and loaded on a Superdex S200 (GE Healthcare) size exclusion column preequilibrated with SEC Buffer (25 mM HEPES pH 8.0, 150 mM NaCl, 1 mM EDTA. 0.1 mM TCEP). PARP-1 was spin-concentrated and flash-frozen in liquid nitrogen.

### Single-molecule microscopy

Single-stranded DNA curtains were assembled in microfabricated flowcells according to published protocols (37–40). Briefly, the template and primer oligonucleotides were annealed by heating to 75°C and cooling at a rate of –1°C min^–1^. Annealed circles were ligated with DNA Ligase (NEB, M0202) for 5 h at room temperature. Long ssDNA molecules were generated in 1× phi29 reaction buffer (NEB, M0269S), 500 μM dCTP and dTTP (NEB, N0446S), 0.2 mg ml^–1^ BSA(NEB, B9000S), 10 nM annealed circles, and 100 nM phi29 DNA polymerase. The mixture was mixed by pipetting and immediately injected on the flowcell and incubated at 30°C for 20–40 min. All microscope experiments were conducted at 37°C. Images were collected on an inverted Nikon Ti-E microscope in a prism TIRF configuration running NIS Elements (AR 4.30.02). Flowcells were illuminated with 488 and 637 nm lasers (Coherent OBIS) split with a 638 nm dichroic mirror (Chroma). Two-color images were recorded by twin electron-multiplying charge-coupled device (EMCCD) cameras (Andor iXon DU897). Uncompressed TIFF stacks were exported from NIS Elements and further analyzed in FIJI (41). Data analysis was performed in MatLab R2019a (MathWorks).

### RPA removal assays

We first generated ssDNA in the flowcells as described previously (39,40). Next, 0.4 nM RPA-GFP was added to Imaging Buffer (40 mM Tris–HCl pH 8.0, 2 mM MgCl_2_, 1 mM DTT, 0.2 mg ml^–1^ BSA, 50 mM NaCl, and 1 mM ATP) and injected at 0.4 ml min^–1^ to tether the ssDNA molecules at a chromium pedestal 13 μm away from the biotinylated anchors. Unbound RPA-GFP washed out with Imaging Buffer. Polθ-h was introduced at the indicated concentration at a flow rate of 0.4 ml min^–1^ and excess helicase flushed from the flowcell. To monitor Polθ-h activity, 2 nM complementary fluorescent oligo (Comp-647N) was added into the flowcell, and flow was stopped (Supplementary Table S6). Other RPA-GFP removal experiments omitted Comp-647N and were monitored by the disappearance of GFP signal. Images with a 50 ms exposure were acquired every 15 seconds using a 14 mW 488 nm laser and a 55 mW 637 nm laser (power measured at the front face of the prism).

To analyze the extent of ssDNA clearance, we isolate a region of interest (ROI) that encompasses the entire Atto647N fluorescent intensity along the DNA at each time point. The length of the ROI is determined by the extent of the ssDNA clearance. The ROI is typically three pixels wide to account for the diffraction-limited signal and any transverse ssDNA motion. The signal intensity across the width of the ssDNA ROI is summed and the resulting signal intensity is fit to a Gaussian function (Supplementary Figure S2B). The full width at half-max (FWHM) of the Gaussian fit at each time point is used to measure the rate and processivity of RPA-GFP clearance. Substituting the Gaussian fit with ether a Heaviside function did not change any of the subsequent results. For differing concentration injections of Polθ-h, foci were counted per unit length of the ssDNA molecule. For experiments that quantified total fluorescence intensity, we measured this intensity along the length of the entire ssDNA and normalized to unit length to correct for heterogeneity in the ssDNA lengths.

### RAD51 removal assays

We first generated RPA-coated double-tethered ssDNA as described above. To assemble RAD51 filaments, 1 μM RAD51(K133R) was injected in Imaging Buffer supplemented with 1 mM CaCl_2_, and flow was stopped for 10 min. Flow was resumed at 40 μl min^–1^ to remove unbound RAD51. Polθ-h was introduced at the indicated concentration and a flow rate of 0.4 ml min^–1^. Because of RAD51′s strand capture activities, we could not use a fluorescent complementary oligo to monitor helicase translocation. Instead, we monitored RAD51(K133R) clearance by adding 2 nM RPA-GFP to the flowcell. At this concentration, RPA cannot readily replace RAD51(K133R) on the ssDNA. Images with a 50 ms exposure are acquired every 15 s using a 40 mW 488 nm laser. We fit the GFP fluorescent intensity to a Gaussian distribution. The FWHM of the Gaussian distribution at each time point measured the extent and rate of RAD51(K133R) clearance. Fluorescent molecules were quantified as described for the RPA clearance experiments described above.

### Polθ-h helicase assays

Short-range Polθ-h helicase activity was measured as described previously (27). Briefly, oligo IF915 was radiolabeled with ^32^P by T4 Polynucleotide Kinase (NEB M0201). IF915 was hybridized with IF916 at a 1:1.2 molar ratio by heating to 95°C and cooled at –1°C min^–1^ in a thermocycler to generate duplex DNA with a 3′ ssDNA overhang. 5–20 nM Polθ-h or Polθ-h(3A) was incubated with 2 nM duplexed oligonucleotides for 10 min at room temperature. Helicase activity was initiated with the addition of 2 mM ATP and 100 nM unlabeled chase IF915 oligonucleotide. Reactions were performed at room temperature for 20 min and quenched with 100 mM EDTA, 0.5% SDS and 0.2 mg ml^–1^ Proteinase K. The reaction was resolved on 15% native PAGE gels.

Polθ-h long-range helicase activity was measured in flowcells containing double-stranded DNA (dsDNA), as used previously for RecQ-family helicases (35,42). The DNA substrate was derived from bacteriophage λ. The *cosL* end was ligated with LAB07 and *cosR* with Lambda Poly-T that produces a 3′-T_78_ overhang (Supplementary Table S6) (35). Polθ-h was injected into the flowcell in Imaging Buffer at 0.4 ml min^–1^. Unbound Polθ-h was washed out and the buffer was switched to Imaging Buffer containing 0.1 nM RPA-GFP at 0.4 ml min^–1^ to fluorescently label exposed ssDNA. The fluorescent intensity of RPA-GFP foci was calculated by averaging the area of a 3 × 3-pixel region of interest. We fluorescently stained DNA with YOYO-1 at the end of the experiment to confirm that RPA-GFP foci localized to DNA ends.

### DNA tethering assays

For single-stranded capture experiments, we first generated ssDNA as described above. 0.4 nM RPA-GFP is added to Imaging Buffer and flown through the flowcell at 0.4 ml min^–1^ to double-tether the ssDNA molecules. Unbound RPA-GFP was flushed out with Imaging Buffer and 1 nM Polθ-h was injected at 0.4 mL min^–1^. To monitor Polθ-h oligo capture, 2 nM noncomplementary fluorescent oligo (Noncomp-647N) was then added to the flowcell (Supplementary Table S6). Binding was monitored by acquiring 50 ms images every 15 s using 14 mW 488 nm laser and 55 mW 637 nm laser.

For double-stranded DNA end bridging experiments, we hybridized λ-phage DNA with LAB07 and Lambda Poly-T oligos by thermal melting and subsequent ligation with T4 DNA Ligase (NEB, M0202) as previously described (Supplementary Table S6) (35). The DNA was fluorescently stained with YOYO-1 to visualize end-tethering.

PARP-1 experiments were carried out in Imaging buffer without BSA. We omitted BSA because it acts as a competitor for PARP-1 activity that inhibits Polθ-h PARylation. First, 5 nM Polθ-h was injected in Imaging Buffer minus BSA. Second, PARP-1 was labeled with an anti-HA primary and goat anti-mouse QDot705 secondary antibodies (ICL RHGT-45A-Z and Thermo Q-11461MP) and injected into the flowcell at a final concentration of 20 nM enzyme (43). To initiate PARylation, we switched to Imaging buffer supplemented with 50 μM NAD^+^. End-tethering was monitored by acquiring 50 ms images every 5 s using a 488 nm laser (14 mW at the front prism face). Negative control experiments either lacked PARP-1 (mock injection) or NAD^+^ (PARP-1 alone). Alternatively, we allowed PARP-1 to autoPARylate before injection into the flowcell. For this experiment, 500 nM PARP-1 was mixed with 4.5 mM NAD^+^ and 500 nM annealed oligos (NJ061 and NJ062) and incubated at 30°C before being diluted before introduction on the flowcell with a final concentration of 20 nM enzyme.

### Ensemble PARylation

We performed Polθ-h PARylation reactions in automodification buffer (30 mM HEPES pH 8.0, 50 mM NaCl, 1.5 mM MgCl_2_, 1 mM DTT) with 1 μM Polθ-h, 500 nM PARP-1, 4.5 mM NAD^+^, 500 nM annealed oligos (NJ061 and NJ062) at 30°C (Supplementary Table S6) (36). Western blots were imaged on an Odyssey imaging system (Licor) with anti-PAR primary and goat anti-mouse IR680 (Millipore Sigma AM80 and Abcam ab216776, respectively). A dT_50_ oligo was radioactively labeled with ^32^P by T4 PNK (NEB M0201). EMSAs were performed in Imaging Buffer at room temperature. Polθ-h ssDNA displacement EMSAs were performed in automodification buffer with 25 nM PARP-1. Protein incubations were performed in low adhesion microcentrifuge tubes (Simport, T330-7LST) to reduce non-specific adsorption of PARylated proteins and associated DNA to the tube walls.

## RESULTS

### Polθ-helicase strips RPA from single-stranded DNA

We purified and confirmed that the Polθ helicase domain (amino acids 1–894, referred to as Polθ-h) assembles into homotetramers via calibrated size exclusion chromatography consistent with previous studies (Figure 1A, Supplementary Figure S1) (44). Next, we monitored single Polθ-h complexes using single-stranded DNA (ssDNA) curtains (Figure 1B) (38,39). In this assay, ssDNA is generated by rolling-circle amplification of a repeating 28-nucleotide minicircle with low structural complexity (45,46). The 5′ end of the primer includes biotin and the resulting ssDNA molecule is immobilized on the surface of a fluid lipid bilayer via biotin-streptavidin interactions. The ssDNA is then extended from the tether point via mild buffer flow.

We first assayed how Polθ-h counteracts RPA-coated ssDNA because RPA inhibits hybridization of heteroduplex oligos during TMEJ (19). We monitored the removal of fluorescent RPA-GFP because multiple fluorescent labeling strategies resulted in hypoactive Polθ-h (Supplementary Figure S2A, D) (47). In this assay, ssDNA curtains are assembled with RPA-GFP. Next, unlabeled Polθ-h is added to the flowcell, and unbound protein is washed out. RPA clearance is observed following injection of fluorescent complementary oligonucleotide that can tile across the ssDNA substrate (Comp-647N) (Figure 1C). Injecting Polθ-h into the flowcell created a punctate pattern with reduced RPA-GFP signal and increased fluorescent oligonucleotide binding. RPA clearance and oligo binding required Polθ-h, suggesting that the helicase clears the ssDNA by removing RPA. Polθ-h cleared RPA along the entire ssDNA molecule, with a slight decrease at the 5′ end due to optical interference from the chromium barrier (Figure 1C, Supplementary Figure S2E).

Next, we quantified RPA removal on double-tethered ssDNA curtains. RPA-ssDNA is tethered to downstream microfabricated chromium features and buffer flow is then stopped to observe protein dynamics in the absence of hydrodynamic force. With Polθ-h and 1 mM ATP, all RPA-free regions expanded with a 3′ to 5′ polarity (*N* = 91 Polθ-h molecules), consistent with other SF2-family helicases (27,48) (Figure 1D). ssDNA without RPA signal was rapidly hybridized by Comp-647N, indicating that Polθ-h created RPA-free regions. Consistent with this observation, RPA-GFP intensity decreased more rapidly in the presence of Polθ-h and 1 mM ATP than the photobleaching-limited signal loss in the negative control experiments without the helicase or with an ATPase-dead Polθ-h (E121A, D216A, and E217A; termed the 3A mutant) (21) (Figure 1E). We also observed small Comp-647N puncta when Polθ-h and/or ATP were omitted from the reaction. These foci were static throughout the experiment and likely represent locations where RPA-GFP is transiently displaced by excess Comp-647N (Supplementary Figure S2F, Supplementary Table S1). To estimate the processivity and rate of Polθ-h translocation, we fit the Comp-647N signal to a Gaussian function and calculated the full-width at half-max for each time point (Supplementary Figure S2B,C). Polθ-h is a processive enzyme, clearing ∼3.9 kilonucleotides (knt; IQR = 2.7–5.1 knt; *N* = 91 Polθ-h molecules) of RPA-coated ssDNA with a median velocity of 63 nt s^–1^ (IQR = 28–117 nt s^–1^, *N* = 91) (Figure 1F, G). Omitting Comp-647N from the reaction did not alter the translocation rate of Polθ-h as measured by RPA-GFP removal (Supplementary Figure S2G). Increasing Polθ-h concentration increased the number of Comp-647N foci per unit length and increased the total Comp-647N fluorescence intensity along the ssDNA substrate (Figure 1H, I, Supplementary Figure S2H, Supplementary Table S2). Increasing Polθ-h(3A) concentration also increased the number of Comp-647N foci on RPA-coated ssDNA curtains. However, these foci did not show time-dependent increases in fluorescence intensity, indicating that Polθ-h(3A) is not translocating on ssDNA to load multiple Comp-647N oligos (Supplementary Figure S2I, J). We conclude that Polθ-h loads at multiple distinct positions along the ssDNA substrate. Increasing Polθ-h concentration did not change the rate of translocation, indicating that each clearance event is likely a single Polθ-h complex (Supplementary Figure S2K). Taken together, we show that Polθ-h is a processive 3′ to 5′ ssDNA motor that uses ATP hydrolysis to strip RPA from ssDNA.

Polθ-h can unwind short duplex DNA molecules and DNA-RNA hybrids with limited processivity (27). Having observed processive ssDNA translocation, we next tested whether Polθ-h is also a processive helicase. We confirmed that our Polθ-h preparation, but not the ATPase-inactive Polθ-h(3A), displayed robust helicase activity on oligonucleotide-length substrates (Supplementary Figure S3A) (27). We then used a double-stranded DNA (dsDNA) substrate with 3′-ssDNA overhangs that mimic TMEJ resection intermediates to explore long-range activity. Helicase activity generates ssDNA that can be monitored via a growing RPA-GFP signal (Supplementary Figure S3B) (35). However, the RPA-GFP intensity did not change when Polθ-h and ATP were added to the flowcell (Supplementary Figure S3C). Although we cannot rule out limited helicase activity below our ∼500 bp resolution, we conclude that Polθ-h is not a processive helicase on dsDNA (25,27,44).

### Polθ-h poorly disassembles RAD51 filaments

In addition to clearing RPA, Polθ has been proposed to antagonize HR by removing RAD51 filaments from ssDNA (21,22). To test this hypothesis, we developed an assay to monitor Polθ-h-dependent RAD51 removal. RAD51 turnover on ssDNA is stimulated by its intrinsic ATPase activity but can be inhibited by adding Ca^2+^ to stabilize the pre-formed filament (49). However, Ca^2+^ also inhibits Polθ-h translocation on ssDNA (Supplementary Figure S4A). Therefore, we used the ATPase-deficient RAD51(K133R) to stabilize RAD51 on ssDNA with ATP and Mg^2+^ in the reaction buffer (Supplementary Figure S4B, C) (50). This mutation disrupts the Walker B ATPase motif, permitting ATP binding but not hydrolysis. We confirmed that RAD51(K133R) rapidly displaces RPA-GFP from ssDNA similarly to wild-type RAD51, albeit with a slightly longer nucleation phase (Supplementary Figure S4D). As expected, RAD51(K133R) filaments are also more stable than WT RAD51 when challenged with RPA-GFP in the presence of Mg^2+^ and ATP (Supplementary Figure S4E). In sum, RAD51(K133R) filaments assemble on ssDNA but remain stable in a buffer that also supports Polθ-h translocation.

**Figure 2.**
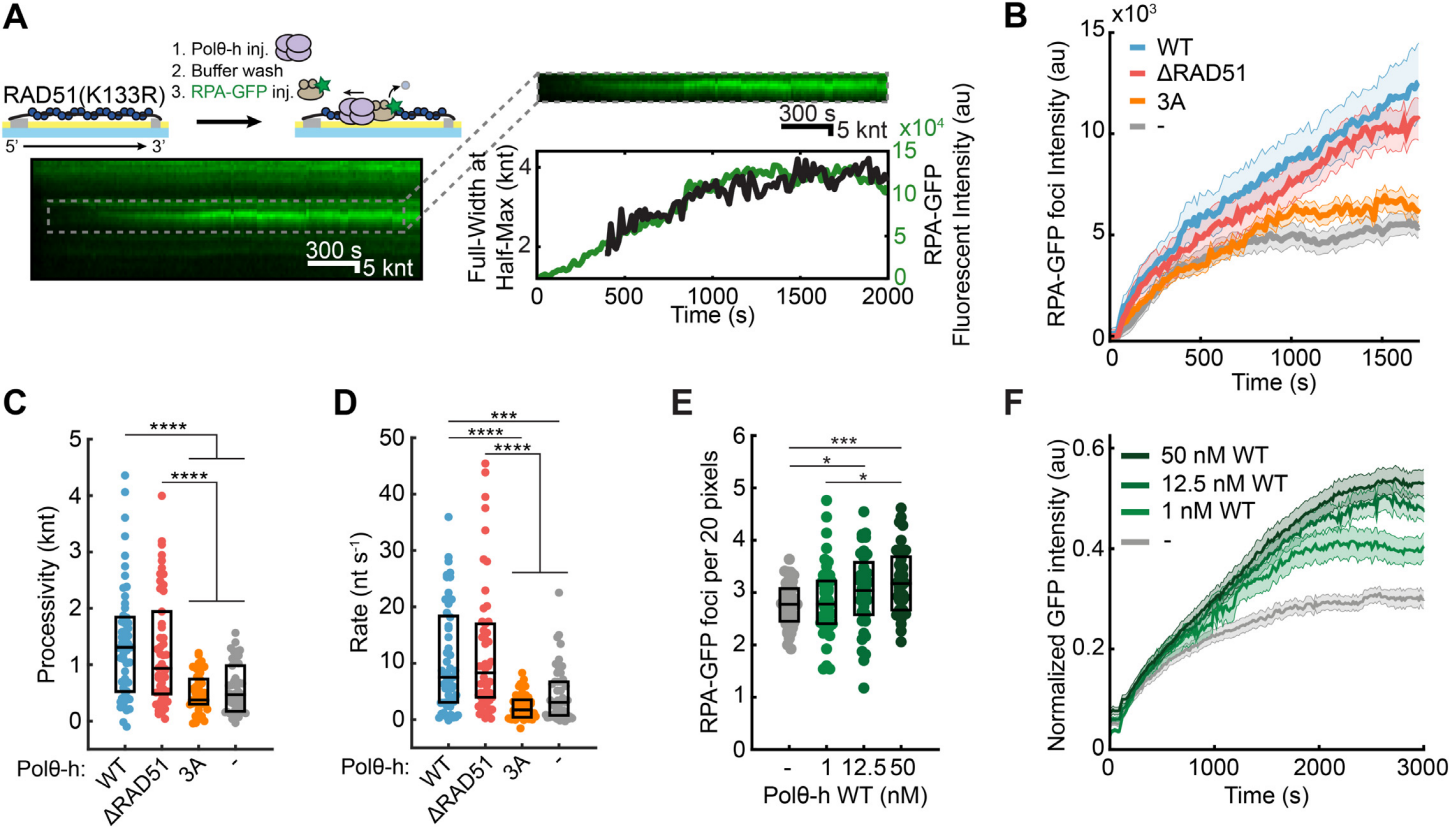
Polθ-h disassembles RAD51 filaments. (**A**) Cartoon, kymograph, and quantification of Polθ-h-mediated removal of RAD51(K133R), as monitored via RPA-GFP binding. (**B**) Quantification of the RPA-GFP foci fluorescent intensity over time. Solid line (average), shading (±SEM). *N* > 36 for all conditions. 1 nM Polθ-h was used, where indicated. (**C**) 1 nM Polθ-h processivity and (**D**) velocity on RAD51(K133R)-coated ssDNA. Box displays median and IQR. *N* > 36 for all conditions. (**E**) Quantification of RPA-GFP foci for each Polθ-h concentration on RAD51(K133R)-coated ssDNA. Box displays mean and S.D. (**F**) Quantification of the total RPA-GFP intensity per DNA molecule at the indicated Polθ-h concentrations. Solid line (average), shading (±SEM). Time is normalized to when Polθ-h enters the flowcell (*t* = 0). 2 nM RPA-GFP is immediately injected after, and flow is stopped at *t* = 100 s. Fluorescent intensity along ssDNA molecules is normalized to the initial RPA-GFP intensity prior to RAD51(K133R) displacement.

We next tested whether Polθ-h can strip pre-formed RAD51(K133R) filaments from ssDNA. We first coated the ssDNA with RAD51(K133R) and then injected Polθ-h with a low concentration of RPA-GFP to visualize any ssDNA that is created during RAD51 removal (Figure 2A). In the presence of Polθ-h, the RPA-GFP puncta were ∼2-fold brighter (*N* = 53) than Polθ-h(3A) and when Polθ-h was omitted (*N* = 41 and *N* = 46, respectively) (Figure 2B). On RAD51(K133R)-coated ssDNA, the median Polθ-h processivity was 1.3 knt (IQR = 0.5–1.9 knt, *N* = 53) and the velocity was 8 nt s^–1^ (IQR = 3–19 nt s^–1^, *N* = 53) (Figure 2C, D, Supplementary Table S3). We also purified a Polθ-h mutant that ablates a putative RAD51 interacting site via five alanine substitutions at positions 861–865, termed Polθ-h(ΔRAD51) (21). Polθ-h(ΔRAD51) processivity and translocation rate and RAD51 removal activity was indistinguishable from wild-type Polθ-h (Figure 2B-D). Processivity was reduced 3-fold and the velocity was 8-fold slower with RAD51(K133R) as compared to RPA. In contrast to the RPA removal reaction, increasing Polθ-h concentration has only modest effects on the number of RPA-GFP foci per ssDNA (Figure 2E, Supplementary Figure S4F, Supplementary Table S4). Increasing the concentration of Polθ-h(3A) did not change the number of foci per RAD51-coated ssDNA, suggesting that these filaments are harder to disassemble than RPA-ssDNA foci (Supplementary Figure S4G, H). The total RPA-GFP fluorescent intensity along the entire ssDNA substrate increased only ∼2-fold above control experiments with Polθ-h(3A) or omitting Polθ-h (Figure 2F). These results indicate that Polθ-h loads at gaps or junctions in the RAD51 filament to partially disassemble stabilized RAD51 filaments.

### PARP-1 reverses Polθ-h-mediated DNA bridges

**Figure 3.**
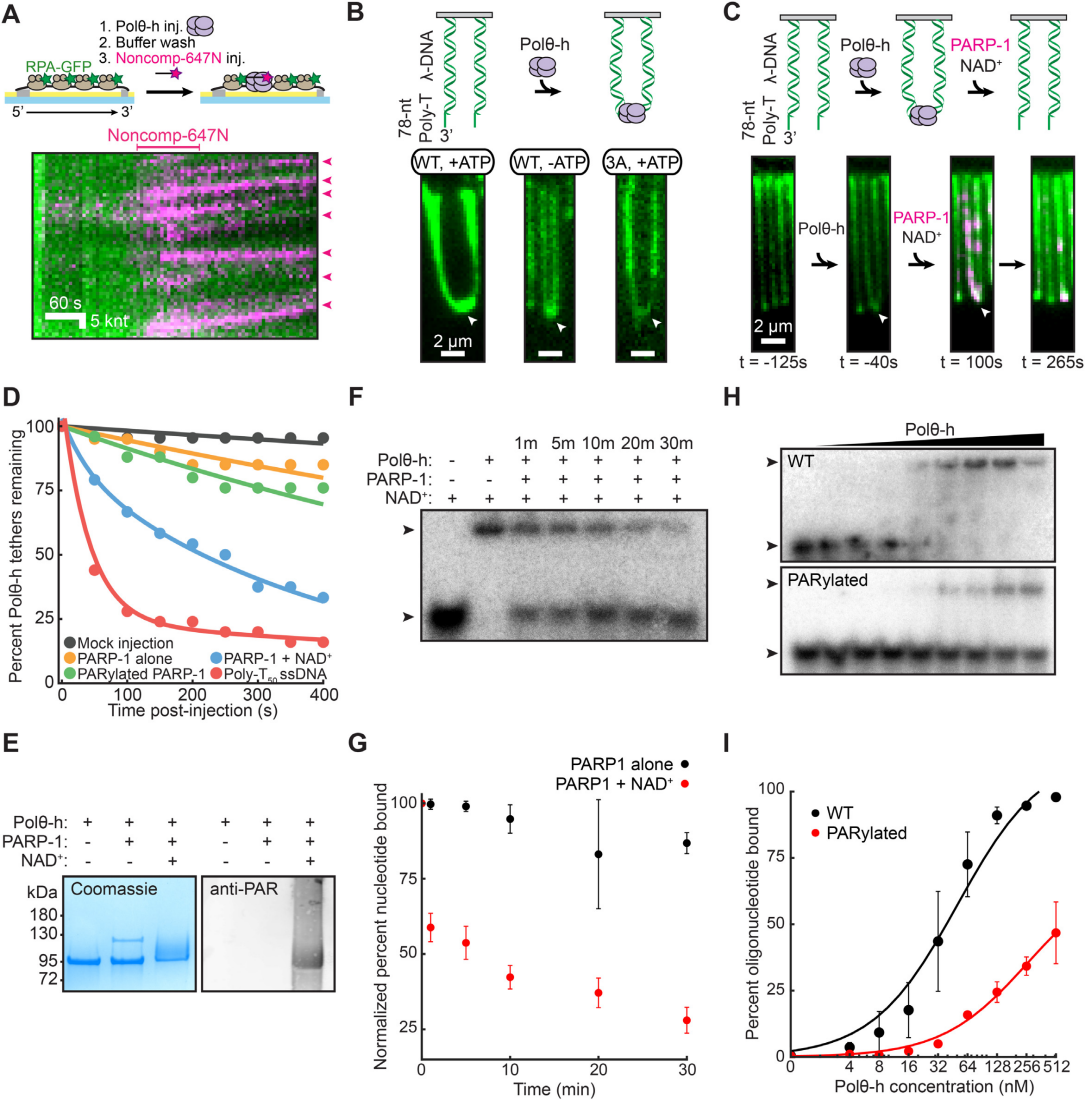
PARP-1 resolves Polθ-h-mediated DNA bridges. (**A**) Cartoon (top) and kymograph (bottom) of 5 nM Polθ-h-mediated tethering of two non-complementary (Noncomp-647N) ssDNA molecules. The long ssDNA is labeled with RPA-GFP (green) and the short non-complementary oligo is labeled with Atto647N (magenta). We observe a processive translocation of Noncomp-647N bound Polθ-h molecules. (**B**) 5 nM Polθ-h tethers DNA molecules with 3′-ssDNA overhangs that mimic resected ends in the presence or absence of ATP. Cartoon of DNA end bridging (top) and images of two tethered λ-phage DNA molecules (bottom). DNA is visualized with YOYO-1 (green). White arrows denote tether points. (**C**) Addition of 100 nM QDot705-labeled PARP-1 (magenta) and NAD^+^ dissociates Polθ-h-mediated bridges. DNA is visualized with YOYO-1 (green). White arrows denote tether points. Time is normalized to when PARP-1 and NAD^+^ enter the flowcell. (**D**) Quantification of lifetimes of Polθ-h-mediated bridges over a variety of conditions. Time is normalized to challenge condition introduction in the flowcell. (**E**) Coomassie (left) and a western blot (right) indicating that Polθ-h is PARylated by PARP-1. (**F**) Electrophoretic mobility shift assay showing that ssDNA-bound Polθ-h dissociates from ssDNA after PARylation. 256 nM prebound Polθ-h and 1 nM radiolabeled ssDNA oligonucleotide were incubated with 25 nM PARP-1 and 4.5 mM NAD^+^ for the indicated times. Arrows indicate unbound and bound oligonucleotide. (**G**) Quantification of (F). Binding normalized to condition without PARP-1. Average of three replicates. Error = SEM. (**H**) WT and PARylated Polθ-h EMSA on 1 nM radiolabeled ssDNA oligonucleotide. Polθ-h concentrations range from 0 to 512 nM. Arrows indicate unbound and bound oligonucleotide. (**I**) Quantification of (H). Fit to hyperbolic equations. Average of three replicates. Error = SEM.

TMEJ initiates after broken DNA ends are resected to reveal ssDNA overhangs (17). Polθ is proposed to bridge these overhangs despite their limited homology. The homotetrameric assembly of the helicase domain may underpin this multivalent DNA binding (44). To test whether Polθ-h can bridge thermodynamically unfavorable microhomologies, we first added Polθ-h to the ssDNA substrate and then flowed in a fluorescent non-complementary oligonucleotide (Noncomp-647N) (Figure 3A). Polθ-h efficiently captured this oligo, indicating that Polθ-h can bridge two ssDNA sequences regardless of homology. Notably, oligo capture required Polθ-h, whereas oligos did not associate with the ssDNA when Polθ-h was omitted (Supplementary Figure S5A). When ATP was added, Polθ-h translocated on the ssDNA with the bound Noncomp-647N oligonucleotides (Figure 3A). Using single-particle tracking of the Noncomp-647N signal, we conclude that the translocation rate is ∼50% decreased, but the processivity is statistically indistinguishable from the RPA-GFP removal activity (Supplementary Figure S5B). We also tested whether Polθ-h can bridge DNA substrates that mimic DNA resection intermediates. We assembled 48 kbp-long dsDNAs with a 3′-T_78_ ssDNA overhang. Adding 5 nM Polθ-h resulted in bridging of adjacent molecules at their free DNA ends (Figure 3B). DNA bridging required Polθ-h but was ATPase independent; omitting ATP or using Polθ-h(3A) produced indistinguishable end-tethered DNAs (Figure 3B). These bridges persisted for the duration of the 10-min imaging experiment. We additionally injected a ∼60-s pulse of 1M NaCl to attempt to dissociate Polθ-h from the ssDNA end. Surprisingly, end-tethering persisted through the high salt wash (Supplementary Figure S5C). Polθ-h also bridges DNAs with long 5′-ssDNA overhangs (T_78_), indicating that this activity is not specific to 3′-overhangs (Supplementary Figure S5D). We also observed DNA bridges when the ssDNA overhangs were pre-loaded with RPA in the presence or absence of ATP (Supplementary Figure S5E).

We reasoned that Polθ-h-DNA bridges must be actively resolved for downstream TMEJ. PARP-1 is an attractive candidate for this activity for three reasons. First, PARP-1 is one of the earliest enzymes to arrive at broken DNA ends and plays a critical role in promoting TMEJ (13,51,52). Second, poly-ADP-ribosylation of client proteins by PARP-1 results in their release from DNA (53–56). Third, a proteomics screen identified the N-terminus of Polθ (i.e. the helicase domain) as a PARylation target (57). Consistent with our hypothesis, adding PARP-1 and NAD^+^ dissolved resected DNA bridges (Figure 3C, D, Supplementary Table S5). Omitting either PARP-1 or NAD^+^ was not sufficient to resolve these DNA bridges alone (Supplementary Figure S5C). Auto-PARylated PARP-1 was also insufficient to resolve these bridges, possibly because this enzyme doesn’t bind the ssDNA junctions (58). Thus, PARP-1 needs to both bind the DNA and localize with Polθ-h to initiate the PARylation reaction. Polθ-h-mediated DNA bridges were also resolved with a 1 μM poly-T_50_ oligonucleotide injection, indicating that other negatively charged polypeptides can recruit Polθ-h away from the DNA bridges. Purified PARP-1 can also PARylate Polθ-h *in vitro*, as indicated by a supershift of the Polθ-h SDS-PAGE band upon incubation with PARP-1 and NAD^+^ (Figure 3E, Supplementary Figure S5F, G) An anti-PAR western blot confirmed that the upshifted Polθ-h band represents a PARylated product.

We further quantify whether PARylated Polθ-h has impaired ssDNA binding relative to the unmodified enzyme using electrophoretic mobility shift assays (EMSAs). Polθ-h was pre-incubated with a radiolabeled dT_50_ oligonucleotide prior to the addition of PARP-1 and NAD^+^. ssDNA-bound Polθ-h is rapidly released from ssDNA upon PARylation (Figure 3F, G). ssDNA remained bound by Polθ-h in the presence of only PARP-1 (Figure 3G, Supplementary Figure S5H). We also changed the order of addition by pre-incubating Polθ-h with PARP-1 and NAD^+^ prior to incubating with a radiolabeled dT_50_ oligonucleotide (Figure 3H). Unmodified Polθ-h had a 39 ± 12 nM ssDNA binding affinity, which closely matches the 30 nM affinity measured via fluorescence anisotropy assays (44). In contrast, PARylated Polθ-h decreased ssDNA affinity at least ten-fold compared to Polθ-h alone (>370 nM) (Figure 3I). Taken together, the single-molecule and ensemble experiments demonstrate that PARP-1 can PARylate Polθ-h and that PARylation reduces the ssDNA binding affinity of Polθ-h.

## DISCUSSION

**Figure 4.**
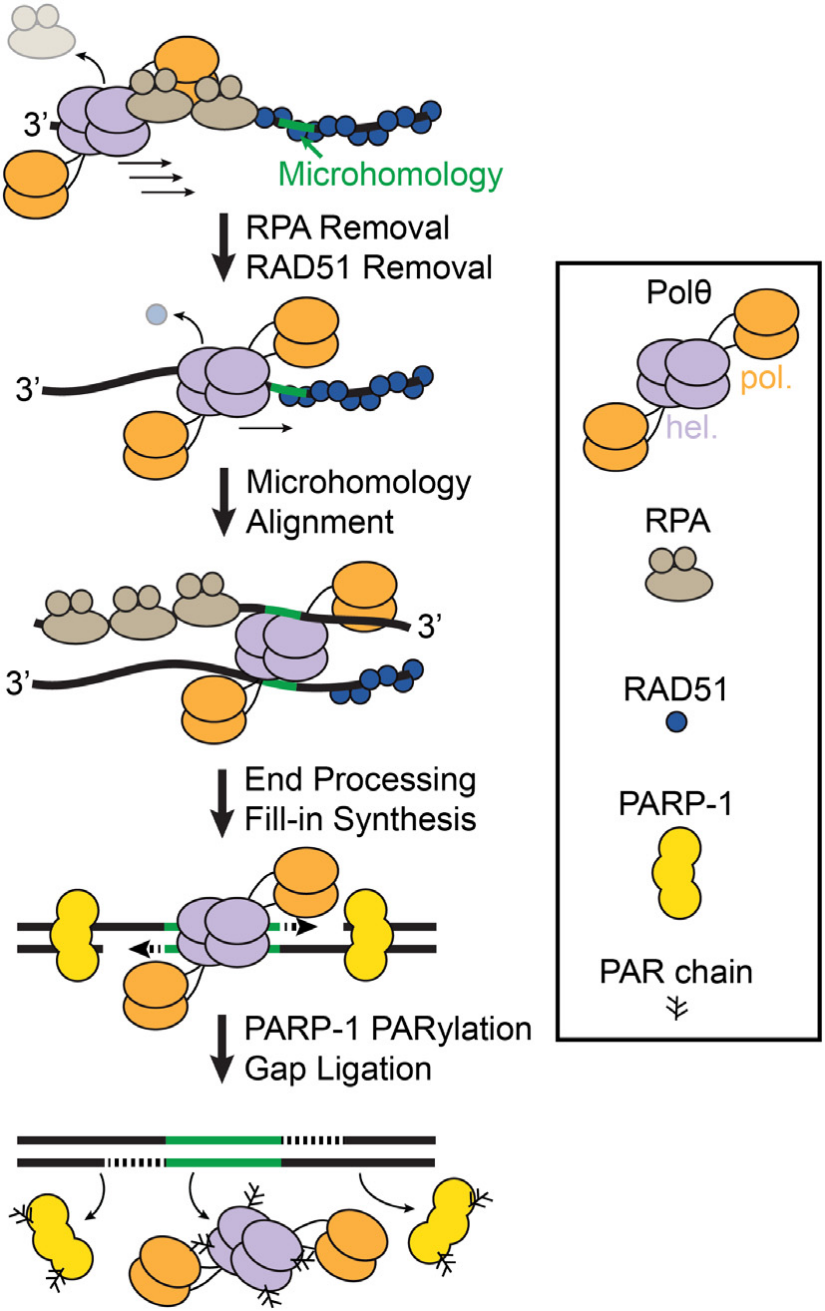
Model of Polθ activities in Theta-mediated end-joining. Polθ-h moves 3′ to 5′ on resected ssDNA to remove RPA and RAD51 and scans for microhomologies (green). 3′ ends are processed and resulting gaps are filled in by the Polθ polymerase domain. PARP-1 PARylates Polθ to remove it from DNA after gap filling.

Figure 4 summarizes our model for how Polθ uses its helicase domain during TMEJ. Polθ encounters RPA-coated ssDNA that is generated during resection. Its helicase domain translocates in a 3′ to 5′ direction to processively remove RPA and other ssDNA-binding proteins from the ssDNA substrate. RPA prevents the hybridization of short microhomologies, so its removal is critical during TMEJ (19). Polθ-h removes RPA over thousands of nucleotides and can also partially disassemble RAD51 filaments *in vitro*. This long-range in vitro translocation activity may be attenuated by the polymerase domain. We used a RAD51 mutant that stabilizes ssDNA filaments in these studies, so these results are likely a lower estimate on Polθ-h's ability to clear wild-type RAD51 filaments. We propose that the helicase domain can load within RPA-coated segments or at RAD51-RPA filament junctions to rapidly remove RAD51 over the tens to hundreds of nucleotides that are required to synapse TMEJ junctions in cells (59–61).

After clearing the ssDNA, Polθ-h bridges two DNA ends. Upon addition of ATP, at least one DNA strand is translocated in relation to the second DNA. A structural Polθ-h domain study suggested that the tetramer may function as a ‘dimer of dimers’, where each half functions independently (44). This dimer-of-dimers arrangement may allow Polθ-h to actively scan for microhomologies by moving a partially complementary overhand along another strand. This may be sufficient for the polymerase domain to extend the microhomologies. Following polymerization, these bridges can be resolved by PARP-1-dependent Polθ PARylation, which reduces the affinity of the enzyme for ssDNA. Removing Polθ may be required for ligases to re-seal the broken DNA breaks.

The robust RPA removal activity that we observed biochemically suggests that RPA clearance is a major target for Polθ-h in cells. Removing RPA increases the accessibility of microhomologies internal from the DNA end and suppresses the RPA-to-RAD51 exchange that precedes the formation of large RAD51 foci in cells (15,59). Polθ ATPase mutants that disrupt the helicase activity also shift the spectrum of TMEJ junctions to microhomologies at the DNA end. We propose that these microhomologies become inaccessible because the helicase domain cannot remove RPA. Polθ-h also loads more efficiently on RPA- versus RAD51-coated ssDNA in a concentration-dependent manner. We conjecture that RPA’s rapid exchange and diffusion on ssDNA may promote Polθ-h's loading relative to RAD51 filaments (62–64). Our observation that Polθ-h has limited RAD51 clearing activity is consistent with previous studies, including reports that RAD51 foci increase in cells that have helicase-dead Polθ (21,22).

The Polθ-h domain is also postulated to be a reverse helicase, or annealase, that can thermodynamically hybridize short microhomologies (26). In this study, we show that Polθ-h can bridge two ssDNA sequences regardless of sequence homology. Based on this result, we suggest that the microhomology selection is mediated by the polymerase domain where Polθ-h initiates a 3′ to 5′ processive ‘microhomology scan’ for the polymerase domain (15,59). Surprisingly, these ssDNA bridges are highly resistant to NaCl, suggesting that additional protein factors are required for their disassembly.

PARP-1 is one of the first DNA damage sensing proteins to localize to DNA damage (65). Polθ recruitment to laser damage is reduced in cells with PARP inhibitors or PARP-1 depletion (22). Our data suggest that PARP-1 may further regulate the activity of Polθ beyond recruitment. PARP-1 binds with high affinity to DSB and ss/dsDNA junctions (66,67). We propose that the PARylation activity on Polθ may aid in regulation and dissociation post-microhomology synthesis. Polθ binds to the resected 3′ ssDNA and processively translocases internally where PARP-1 then potentially PARylates Polθ. This may function in increasing the access to the polymerized DNA for ligation by the LIG3-XRCC1 complex (68). Additionally, PARylation may aid in the iterative microhomology selection and multiple rounds of DNA synthesis via regulation of Polθ DNA-binding (15,69). We also do not rule out that PARylation by PARP-1 may inhibit Polθ DNA binding to favor more accurate forms of repair. Together, this work shows that Polθ plays multiple roles in mediating end-joining at DSBs in NHEJ/HR-deficient cancers and reiterates the importance of understanding the mechanistic functions of Polθ as a promising therapeutic target (12,30–32).

## DATA AVAILABILITY

All data in the manuscript or the supplementary material is available upon request.

## Supplementary Material

gkac119_Supplemental_File
