# Supplementary material for: Polymerase theta-helicase promotes end joining by stripping single-stranded DNA-binding proteins and bridging DNA ends
Source: Nucleic Acids Res. 2022 Mar 31;50(7):3911–21. doi: 10.1093/nar/gkac119 (PMC9023281; doi:10.1093/nar/gkac119)
Supplement: gkac119_Supplemental_File [file gkac119_supplemental_file.pdf]

SUPPLEMENTAL MATERIAL

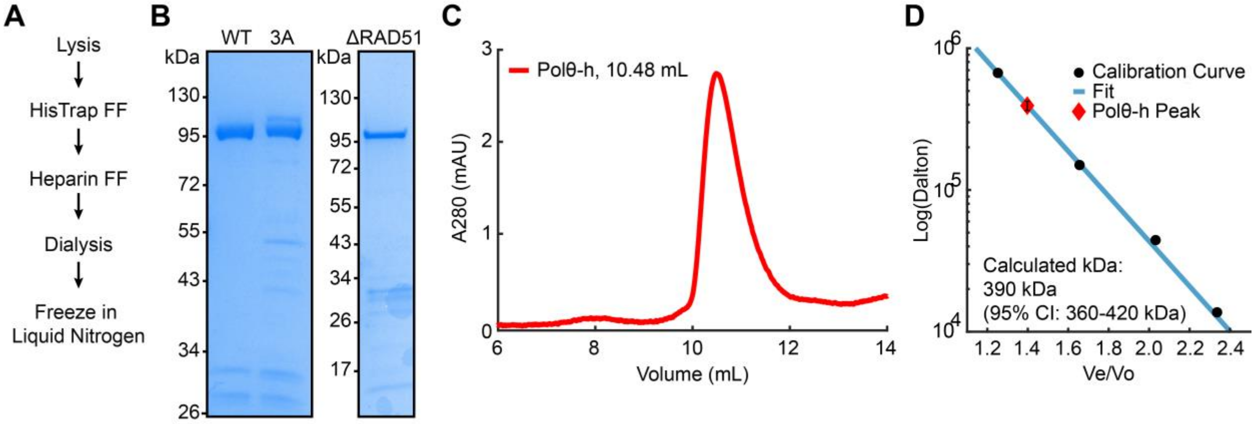

Figure S1: Polθ-h purification and analysis. (A) Schematic of the Polθ-h purification protocol. **(B)** SDS-PAGE gel of purified Polθ-h(WT), Polθ-h(3A), and Polθ-h(ΔRAD51). The expected molecular weight is 99 kDa. **(C)** Superdex-200 chromatogram of recombinant homotetrameric Polθ-h. **(D)** Calculated molecular weight compared to a molecular weight standard (Sigma-Aldrich, 69385). Error bar within the diamond denotes a 95% confidence interval (CI).

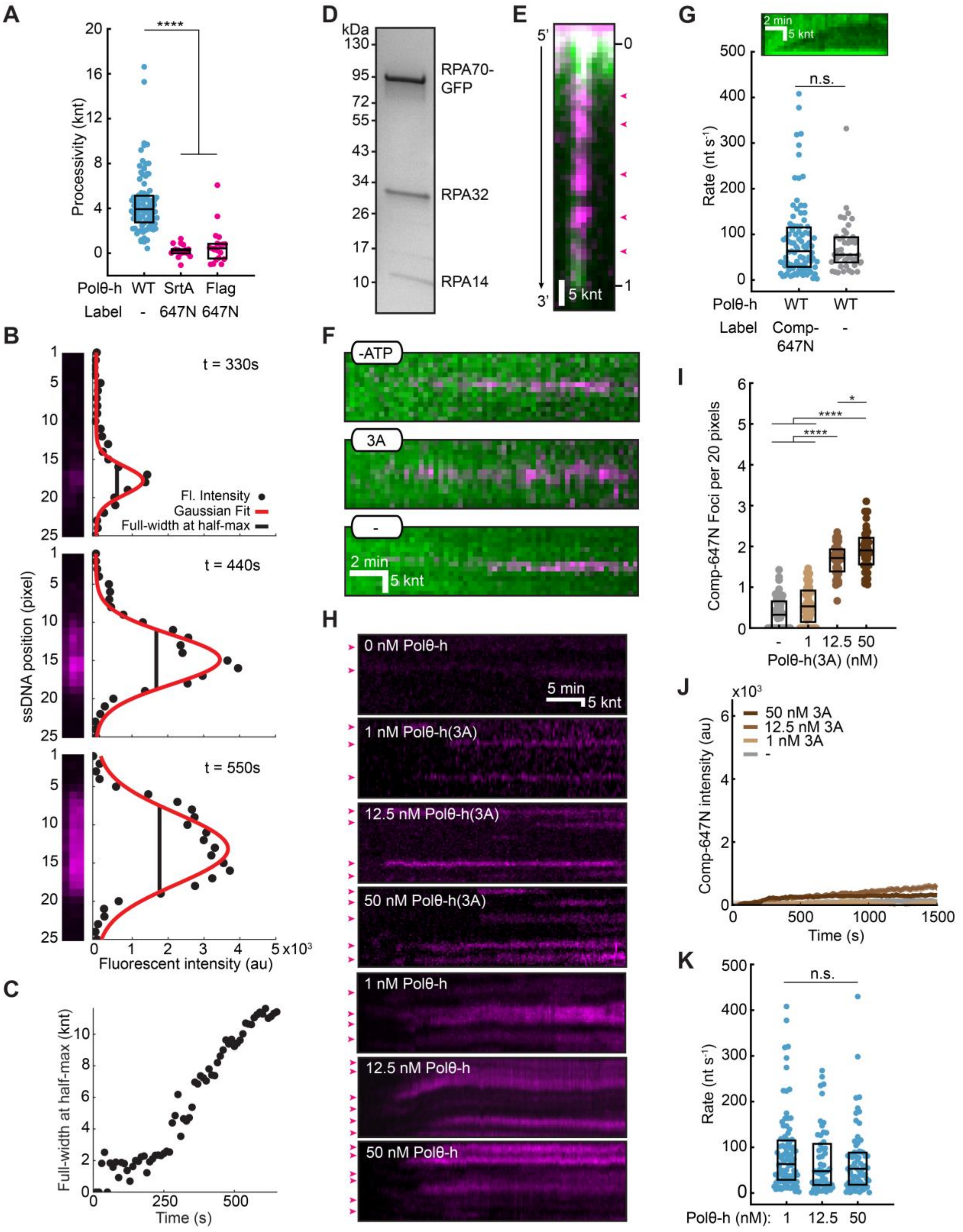
A
Processivity (knt)
****
Polθ-h WT SrtA Flag
Label - 647N 647N
B
t = 330s
t = 440s
t = 550s
Fl. Intensity
Gaussian Fit
Full-width at half-max
ssDNA position (pixel)
Fluorescent intensity (au)
C
Full-width at half-max (knt)
Time (s)
D
kDa
RPA70-GFP
RPA32
RPA14
E
5'
3'
5 knt
F
-ATP
3A
-
2 min
5 knt
G
2 min
5 knt
n.s.
Rate (nt s^-1)
Polθ-h WT WT
Label Comp-647N -
H
0 nM Polθ-h
5 min
5 knt
1 nM Polθ-h(3A)
12.5 nM Polθ-h(3A)
50 nM Polθ-h(3A)
1 nM Polθ-h
12.5 nM Polθ-h
50 nM Polθ-h
I
Comp-647N Foci per 20 pixels
Polθ-h(3A) (nM)
J
50 nM 3A
12.5 nM 3A
1 nM 3A
Comp-647N intensity (au)
Time (s)
K
n.s.
Rate (nt s^-1)
Polθ-h (nM): 1 12.5 50

Figure S2: Fluorescent analysis of Polθ-h variants and their translocation activities. (A) We attempted to fluorescently label Polθ-h via sortase-mediated transpeptidation of the N-terminus or the linker region (C-terminus). N-terminal labeled Polθ-h was completely inactive. C-terminal Sortase (SrtA) or Flag epitope labeled Polθ-h constructs (1 nM) showed decreased processivity relative to WT Polθ-h on RPA coated ssDNA. Therefore, we focused on the WT Polθ-h for this study. Box displays median and IQR. **(B)** Summary of how Polθ-h translocation activity was analyzed via fluorescent proxies. Individual time points of fluorescent oligonucleotides hybridizing to the DNA (left) and resulting fluorescent intensity and Gaussian fit (right). **(C)** A plot of the full-width at half-max of this fit as a function of time. **(D)** SDS-PAGE gel of recombinantly purified RPA-GFP. Expected molecular weights are 97, 29, and 14 kDa. **(E)** Analysis of Polθ-h RPA removal locations on single-tethered ssDNA curtains. Due to heterogeneity in their length, ssDNAs are normalized to unit length in a 5' to 3' direction. Magenta arrows show oligonucleotide foci that denote RPA removal activity. **(F)** Kymographs of 1 nM Polθ-h-mediated RPA-GFP removal controls. ATP and hydrolysis activity are both required to see robust RPA removal. **(G)** Kymograph (top) and rate quantification (bottom) of 1 nM Polθ-h-mediated RPA-GFP removal in the absence of Comp-647N. **(H)** Kymographs of concentration dependent Polθ-h oligonucleotide foci (magenta arrows) on RPA-GFP ssDNA curtains. **(I)** Quantification of fluorescent oligonucleotide foci for each Polθ-h(3A) concentration on RPA-coated ssDNA. Box displays mean and S.D. **(J)** Fluorescent oligonucleotide intensity across each ssDNA for each Polθ-h(3A) concentration. Solid line (average), shading (±SEM). **(K)** Polθ-h translocation rate for three different Polθ-h concentrations on RPA-GFP ssDNA molecules.

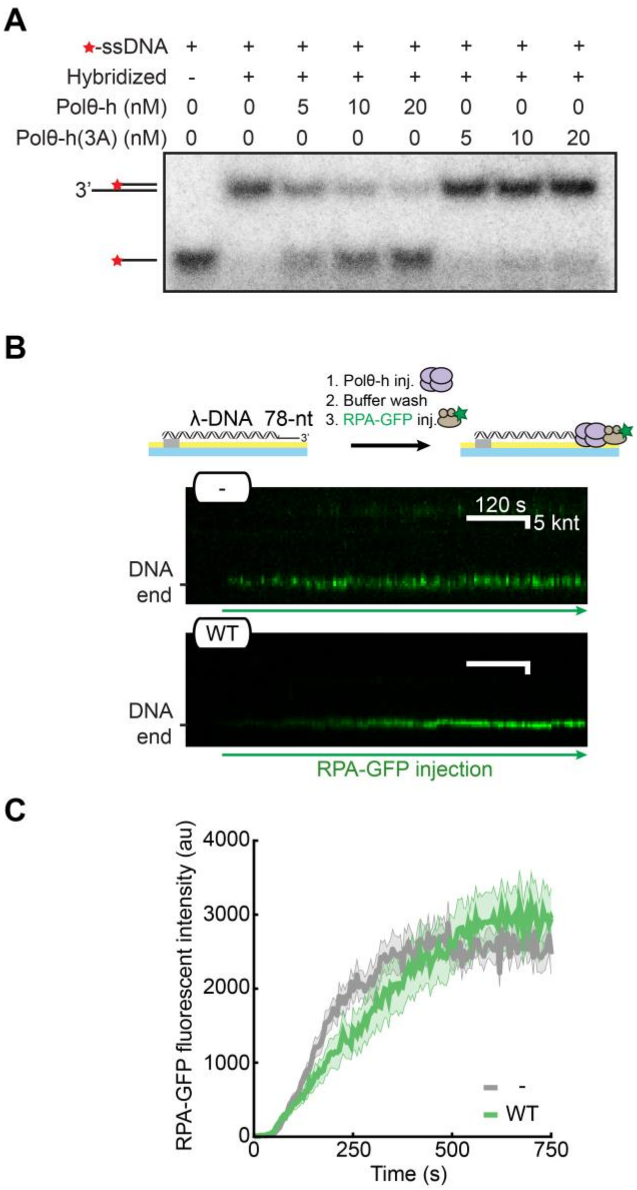

Figure S3: Polθ-h is not a processive dsDNA helicase. (A) Gel-based assay showing short range Polθ-h helicase activity. Helicase activity is abrogated with the 3A mutations. **(B)** Cartoon and kymographs of pre-resected helicase assay substrate in the presence and absence of 1 nM Polθ-h WT. **(C)** RPA-GFP foci fluorescent intensity over time. WT (N=27) and without Polθ-h (-) (N=43).

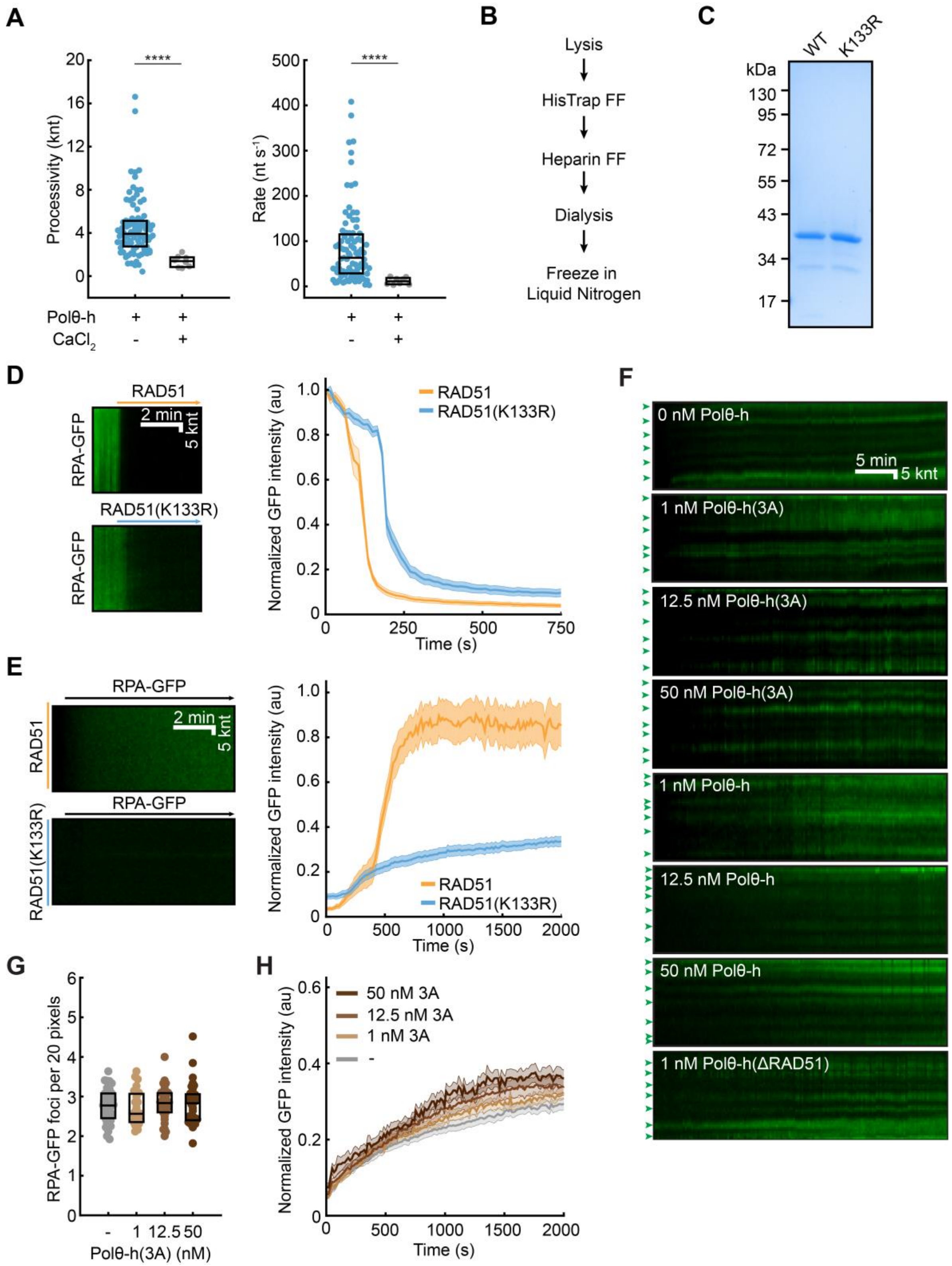
A
Processivity (knt)
Rate (nt s-1)
****
Polθ-h
CaCl2
B
Lysis
HisTrap FF
Heparin FF
Dialysis
Freeze in Liquid Nitrogen
C
WT
K133R
kDa
130
95
72
55
43
34
17
D
RAD51
RAD51(K133R)
RPA-GFP
2 min
5 knt
Normalized GFP intensity (au)
Time (s)
E
RPA-GFP
RAD51
RAD51(K133R)
F
0 nM Polθ-h
5 min
5 knt
1 nM Polθ-h(3A)
12.5 nM Polθ-h(3A)
50 nM Polθ-h(3A)
1 nM Polθ-h
12.5 nM Polθ-h
50 nM Polθ-h
1 nM Polθ-h(ΔRAD51)
G
RPA-GFP foci per 20 pixels
Polθ-h(3A) (nM)
H
50 nM 3A
12.5 nM 3A
1 nM 3A
Normalized GFP intensity (au)
Time (s)

Figure S4: Characterization of RAD51(K133R) and its exchange with RPA. (A) Polθ-h is unable to remove RPA-GFP in the presence of 5 mM $CaCl_2$. Boxes display the median and IQR. **(B)** Schematic of the RAD51 purification protocol. **(C)** SDS-PAGE gel of recombinant WT RAD51 and RAD51(K133R). Expected molecular weights are 37 kDa. **(D)** Kymographs of RAD51 dependent removal of RPA-GFP (left). Quantification of normalized RPA-GFP fluorescence over time (right). We analyzed 25 DNA molecules for both WT RAD51 and RAD51(K133R). **(E)** RAD51(K133R) is resistant to replacement by RPA as compared WT RAD51. Left: kymographs of RAD51 filament turnover as monitored by RPA-GFP. Right: quantification of the normalized RPA-GFP fluorescence (N=25 for both conditions). **(F)** Kymographs of RPA-GFP foci (green arrows) on RAD51(K133R) ssDNA curtains at the indicated Polθ-h concentrations. **(G)** Quantification of RPA-GFP foci for each Polθ-h(3A) concentration on RAD51(K133R)-coated ssDNA. Box displays mean and S.D. **(H)** RPA-GFP intensity across each RAD51(K133R)-coated ssDNA for each Polθ-h(3A) concentration. Solid line (average), shading (±SEM).

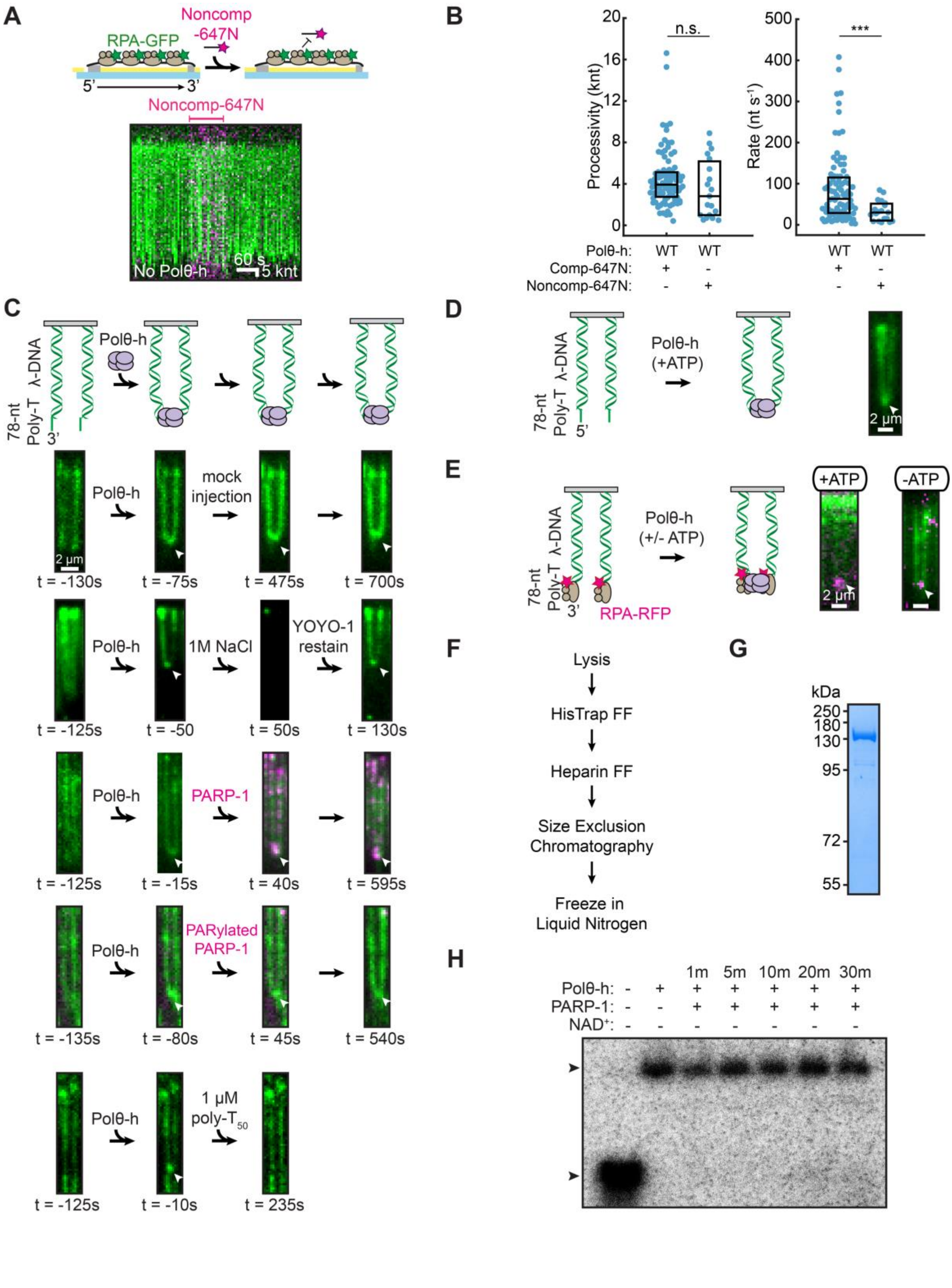

A
Noncomp
-647N
RPA-GFP
5' 3'
Noncomp-647N
60 s
5 knt
No Polθ-h
B
n.s.
Processivity (knt)
***
Rate (nt s-1)
Polθ-h: WT WT WT WT
Comp-647N: + - + -
Noncomp-647N: - + - +
C
78-nt Poly-T λ-DNA
3'
Polθ-h
mock injection
2 µm
t = -130s t = -75s t = 475s t = 700s
1M NaCl
YOYO-1 restain
t = -125s t = -50 t = 50s t = 130s
PARP-1
t = -125s t = -15s t = 40s t = 595s
PARylated PARP-1
t = -135s t = -80s t = 45s t = 540s
1 µM poly-T50
t = -125s t = -10s t = 235s
D
78-nt Poly-T λ-DNA
5'
Polθ-h (+ATP)
2 µm
E
78-nt Poly-T λ-DNA
3'
RPA-RFP
Polθ-h (+/- ATP)
+ATP
-ATP
2 µm
F
Lysis
HisTrap FF
Heparin FF
Size Exclusion Chromatography
Freeze in Liquid Nitrogen
G
kDa
250
180
130
95
72
55
H
1m 5m 10m 20m 30m
Polθ-h: - + + + + + +
PARP-1: - - + + + + +
NAD+: - - - - - - -

Figure S5: DNA bridging requires Polθ-h and is ATP independent. (A) Cartoon and kymograph of noncomplementary-647N (magenta) oligonucleotide injection in the absence of Polθ-h. ssDNA is bound with RPA-GFP (green). **(B)** Comparison of processivity and rates of Polθ-h translocation measured by Comp-647N backfill or Noncomp-647N binding directly to Polθ-h. **(C)** Polθ-h tethering of two dsDNA ends persists for > 10 minutes and is resistant to 1M NaCl, addition of 100 nM QDot705-labeled PARP-1 (magenta) without NAD^+, or 100 nM autoPARylated PARP-1. Polθ-h tethering is dissociated by injection of 1 µM Poly-T_{50} ssDNA. The dsDNA substrate is visualized with the intercalating dye YOYO-1 (green). White arrows denote the DNA tether location. Time is normalized to the injection point. **(D)** Polθ-h tethers two 5' ssDNA overhangs. **(E)** Polθ-h tethers two 3' ssDNA overhangs preincubated with RPA-RFP in the presence or absence of ATP. **(F)** Schematic of the PARP-1 purification protocol. **(G)** SDS-PAGE gel of recombinantly purified PARP-1. N-terminal expression tags remain attached. Expected molecular weight is 129 kDa. **(H)** Prebound Polθ-h and radiolabeled ssDNA oligonucleotide were incubated with PARP-1 without NAD^+ over time.

Table S1: **Polθ-h velocity and processivity on RPA-ssDNA**

Polθ-h	Nucleotide	Processivity, knt (IQR)	Relative processivity decrease	Rate, nt s^{-1} (IQR)	Relative rate decrease	N (foci)
WT	ATP	3.9 (2.7-5.1)	-	63 (28-117)	-	91
WT	-	0.6 (0.1-1.2)	7x	4 (2-12)	16x	57
3A	ATP	0.3 (0.1-0.5)	13x	1 (0.5-4)	63x	46
-	ATP	0.4 (0.1-0.5)	10x	2 (0.2-5)	32x	55
WT	ATP / Ca^{2+}	1.4 (0.8-1.8)	3x	11 (4-20)	6x	8
SrtA-647N	ATP	0.2 (0-0.4)	20x	5 (0-10)	13x	18
Flag-647N	ATP	0.4 (-0.5-0.8)	10x	10 (0-42)	6x	20

Table S2: **Quantification of Polθ-h foci on RPA-ssDNA**

Polθ-h	Concentration, nM	Average foci per 20 pixels (S.D.)	N (ssDNA molecules)
WT	50	3.8 (0.71)	38
WT	12.5	2.7 (0.83)	36
WT	1	1.0 (0.54)	35
3A	50	1.9 (0.50)	36
3A	12.5	1.7 (0.40)	31
3A	1	0.56 (0.45)	41
-	0	0.37 (0.39)	46

Table S3: Polθ-h velocity and processivity on RAD51(K133R) filaments

Polθ-h	Nucleotide	Processivity, knt (IQR)	Relative processivity decrease	Rate, nt s^{-1} (IQR)	Relative rate decrease	N (foci)
WT	ATP	1.3 (0.5-1.9)	-	8 (3-19)	-	53
ΔRAD51	ATP	1.0 (0.5-2.1)	1x	7 (4-16)	1x	49
3A	ATP	0.4 (0.3-0.6)	3x	2 (0-4)	4x	41
-	ATP	0.5 (0.2-1.0)	3x	3 (1-7)	3x	36

Table S4: Quantification of Polθ-h foci on RAD51(K133R)-ssDNA

Polθ-h	Concentration (nM)	Average foci per 20 pixels (S.D.)	N (ssDNA molecules)
WT	50	3.3 (0.63)	38
WT	12.5	3.0 (0.75)	40
WT	1	2.9 (0.70)	43
3A	50	2.8 (0.53)	34
3A	12.5	2.8 (0.40)	32
3A	1	2.7 (0.43)	27
-	0	2.8 (0.41)	44

Table S5: Quantification and dissolution of Polθ-h mediated tether events

Condition	Polθ-h tethers remaining 400s post-injection	Percentage	Half-life
Mock injection	21/22	95%	>400s
PARP-1 alone	17/20	85%	>400s
PARylated PARP-1	19/25	76%	>400s
PARP-1 + NAD^+	8/24	33%	210s
Poly-T_{50} ssDNA	4/25	16%	50s

Table S6: **Oligonucleotides used in this study.**

Oligonucleotide	Sequence (5' to 3')
Template	/Phos/AG GAG AAA AAG AAA AAA AGA AAA GAA GG
Primer	/Biosg/TC TCC TCC TTC T
Comp-647N	/AT647/AGG AGA AAA AGA AAA AAA GAA AAG AAG G
Noncomp-647N	/AT647/TCC TCT TTT TCT TTT TTT CTT TTC TTC C
Poly-T_{50}	T_{50}
NJ061	GGG TTG CGG CCG CTT GGG
NJ062	CCC AAG CGG CCG CAA CCC
LAB07	/Phos/AGG TCG CCG CCC/BioTEG/
Lambda Poly-T	/Phos/GGG CGG CGA CCT T_{78}
IF724	GGA GAA TTC CGA ACT GGG AGG ACC CAG ATC TGT CAT ACG C
IF725	GCG TAT GAC AGA TCT GGG TCC TCC CAG TTC GGA ATT CTC C
IF733	CTC CTA CAA GTG CTG GGG CGA CTC TTG TGG CAG
IF734	GGA ATG GTG GTT GTG GCT GCA TTA CAT ATG CTG GGA GAC TC
IF915	CTG TCC TGC ATG ATG
IF916	CAT CAT GCA GGA CAG TCG GAT CGC AGT CAG
IF926	GCC GCG GCC GGC AGA AAA GGT TTA ACT GAA AGG GAA GCA GCA GCC C
IF927	CGC GGC TCG CAT ATT GCG ACG TTC TTC AAC TGC TTC CTC TTC C
